# The effects of attitudes, norms, and perceived control on the adaptation of elderly individuals and individuals with chronic health conditions to heatwaves

**DOI:** 10.1186/s12889-024-17712-w

**Published:** 2024-01-22

**Authors:** Kaddour Mehiriz

**Affiliations:** Doha Institute for Graduate Studies, Zone 70, Al Tarfa Street, Doha, Al Daayen PO BOX: 200592, Qatar

**Keywords:** Heatwave protection behaviour, Theory of planned behaviour, Social norms, Attitudes, Perceived control, Intentions

## Abstract

**Background:**

In this study, the theory of planned behaviour (TPB) was used to examine the determinants of the heat protection intentions and actions of elderly individuals and individuals with chronic health conditions. This is an important topic as understanding the motivations for adapting behaviours to heatwaves can inform the design of warning systems and awareness campaigns by public health authorities to mitigate the adverse effects of weather hazards on health.

**Methods:**

Three phone surveys were conducted in 2015 and 2016 to collect data on a large sample of individuals with increased vulnerability to heatwaves in the city of Longueuil, Canada. Prospective and panel fixed effects logit models for ordinal variables were used to analyse the factors that influenced heat protection intentions and actions.

**Results:**

Attitudes, norms, and perceived control have positive effects on intentions to adopt heatwave protection actions and intentions on the effective adoption of these preventive measures. The hypothesis according to which perceived control moderates the effect of attitudes and norms on intentions is rejected. In addition, the results suggest that elderly individuals are less likely than individuals in other age groups to adopt heat protection actions. Health conditions related to vulnerability to hot weather do not seem to significantly improve the adoption of heat protection behaviours.

**Conclusions:**

The adoption of heatwave protection actions can be improved by public health interventions that influence attitudes and social norms related to heat protection behaviours and facilitate their adoption.

## Study background

Heatwaves such as those that occurred in France (2003) and the Canadian provinces of Quebec (2010) and British Columbia (2021) are associated with an excess of mortality and morbidity, notably among elderly individuals, individuals with chronic health conditions and disadvantaged social groups [1–3]. As the severity and frequency of heatwaves are increasing due to global warming, heat warning systems and awareness and prevention campaigns are among the main policy tools used by public health authorities to mitigate their adverse effects [3, 4]. These interventions are based on the assumption that when individuals are provided with relevant and accurate information on heat risks and safety tips, they improve their adaptation to heatwaves [3]. The design of heatwave warnings and awareness campaigns would thus benefit from insight into the factors that motivate individuals to adapt their behaviour to these weather hazards [5].

Studies of the determinants of heat prevention behaviours have examined the effects of sociodemographic characteristics such as age, sex and health conditions [4, 6, 7]. Other studies have used sociocognitive models of human behaviour, notably the health belief model [8], protective action decision model [9], theory of planned behaviour [10] and protection motivation theory [11]. These models share the assumption that individuals strive to survive by adopting protective behaviours, including actions to reduce environmental threats [11]. Their interest lies in their parsimony, that is, their capacity to propose for empirical testing a small number of factors that are presumed to explain heat protection behaviours [12]. The results of studies using these frameworks suggest that the adoption of heatwave protection actions is positively associated with expected benefits, attitudes and social norms [8, 10, 13]. However, mixed results have been found regarding the effects of perceived risks and self-efficacy [4, 9, 10].

**Fig. 1:**
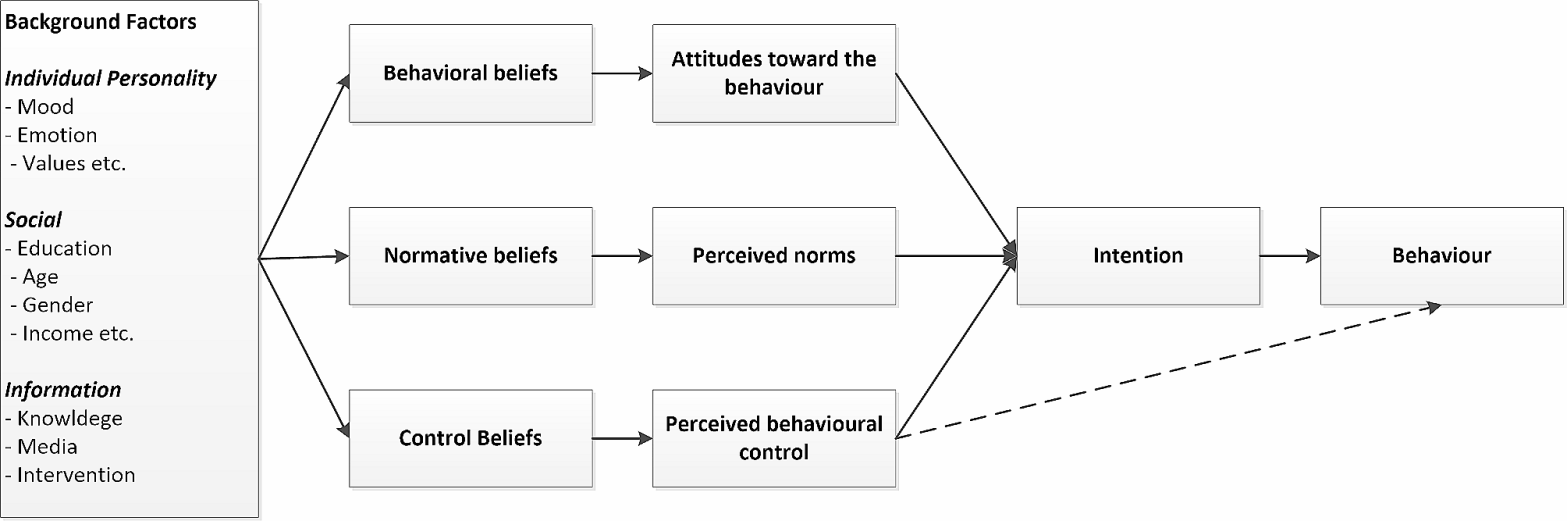
Theory of planned behaviour [15]

In this study, the theory of planned behaviour (TPB) is used to analyse the factors that influence the adoption of five heat protection behaviours commonly recommended by public health authorities and experts [14–17]. These include drinking more water, reducing physical effort, taking cool showers and baths, visiting cool or air-conditioned places and using air conditioning systems. As Fig. 1 shows, the TPB presumes that the adoption of heat protection actions depends on individuals’ intentions to adopt these actions and their perceived control in implementing them. Intentions depend on attitudes, perceived social norms and perceived control corresponding to heatwave protection behaviours. To trace the causal mechanisms of protective actions, the TPB assumes that attitudes, norms, and perceived control are forged by beliefs that are rooted in individuals’ personalities and socioeconomic and demographic characteristics.

Although the TPB is widely used to explain health protection and pro-environmental behaviours [16, 17], it has rarely been used to predict the actions taken by individuals to protect themselves from heatwaves. As demonstrated by McEachan et al. (2011) in their meta-analysis of the TPB, the explanatory capacity of this theory varies across behaviours and populations [18]. This heterogeneity underlines the importance of conducting studies to assess the capacity of the TPB to explain individuals’ decisions to protect themselves from heatwaves. In addition to their contribution to knowledge, the results of these studies can be used to enhance the design of policy initiatives to improve individuals’ adaptation to heatwaves, as is the case in other public health fields [19, 20].

With respect to the TPB, the main objective of this study is to provide evidence related to the following research questions:

- What are the effects of attitudes, prescriptive norms and perceived control on the intention to adopt heat protection actions?
- Does perceived control moderate the effect of attitudes and prescriptive norms on the intention to adopt heat protection actions?
- Are individuals’ intentions and perceived control associated with their heat protection actions?

Although not among the primary objectives of this study, the influence of vulnerability factors (e.g., age and chronic health conditions) on heat protection behaviours are also explored in this article.

The main contribution of this paper to the small body of studies that have used the TPB to analyse adaptation to heatwaves is twofold: we use robust designs to mitigate consistency and endogeneity biases, and we test whether perceived control moderates the effect of attitudes and norms on intentions. For the first contribution, most studies that have adopted the TPB use cross-sectional designs and, consequently, are vulnerable to consistency and endogeneity biases [21]. Consistency bias is present when all the constructs of the TPB are measured simultaneously. In their efforts to avoid cognitive dissonance, respondents strive to impose coherence between their reported behaviours, intentions, attitudes and perceived norms and control, leading to inflated coefficients of association between the components of the TPB. Moreover, in the TPB, intentions are supposed to impact the future rather than current or past behaviours; accordingly, they should be measured before behaviours [22]. To address these problems, this study used a prospective design that involved collecting data on heat protection behaviours one month after data were collected on presumed drivers of behaviour, as explained in the methods section. [19, 20]. Regarding the endogeneity issue, it is plausible that in addition to the factors advanced by the TPB, other variables have direct effects on intentions and behaviours. Unfortunately, most studies do not adequately control for these factors when testing the TPB [10]. In this study, the fixed effect panel model for ordinal variables was used to test the effects of attitudes, norms, and perceived control on intentions, allowing for the control of constant unobservable confounding factors. This design provides a consistent foundation for inferring causal relationships between these variables in comparison with cross-sectional and prospective designs.

The second contribution of this paper is its test of whether perceived control moderates the effects of attitudes and perceived norms on intentions. In some versions of the TPB [14], the effect of attitudes on intentions is assumed to be greater for individuals with high perceived control. Individuals with high perceived control are also assumed to pay less attention to social norms in their behaviours. The moderating effect of perceived control remains largely underinvestigated in empirical studies of the TPB [14].

## Methods

### Study variables

This study used the TPB to predict five heat protection behaviours: drinking water, using air conditioners, taking cool showers and baths, reducing physical effort, and visiting cool or air-conditioned places. These behaviours were chosen because they are frequently recommended by public health authorities and experts to protect against heatwave risks [3, 23].

Attitudes were measured by the perceived utility of the five behaviours to protect respondents’ health from the risks of heatwaves. The utility of each behaviour was measured on a five-point scale (1 = completely useless to 5 = completely useful). Two items were used to measure prescriptive norms. The first item measured on a five-point scale the extent to which respondents agreed with the statement that most of their significant persons thought that they should take actions to protect themselves from heatwaves (1 = totally disagree to 5 = totally agree). The second item measured, on the same scale, the likelihood that most of their significant persons would advise them to adopt heat protection actions (1 = very improbable to 5 = very probable). The average score of the two items was used in the regression analyses. For perceived control, the respondents were asked how easy or difficult it was for them to adopt each of the five heat protection behaviours in question (1 = very difficult to 5 = very easy). To measure intentions, the respondents were asked to indicate their intention to adopt each of the recommended behaviours in the next heatwave (1 = very unlikely to 5 = very likely).

Data on the adoption of heat protection behaviours were collected immediately after the 2015 heatwave from July 27 to 29. This method was expected to yield more accurate data on heat protection behaviours than the commonly used method of asking individuals to report their behaviours during heatwaves in general [24, 25]. The study participants were asked to indicate the frequency of drinking water and exerting physical effort during this heatwave (1 = much less often than usual to 5 = much more often than usual). With regard to cool showers and baths, frequenting cool or air-conditioned places, and using air conditioners, the respondents were first asked about whether they had adopted each behaviour. Those who answered affirmatively were then asked to indicate on a five-point scale their frequency of adopting this heat protection action (1 = much less often than usual to 5 = much more often than usual). For each behaviour, the answers to these two questions were merged to create a new variable that took the value of 0 if the respondent had not adopted the behaviour at all and 5 if the respondent had adopted the behaviour much more often than usual.

### Study sample

This study is part of a research project on the effects of an automated weather warning system for people vulnerable to heat and smog that was conducted in the city of Longueuil, Canada, between 2015 and 2017. This study was approved by the ethics committee of the National Institute for Scientific Research (CER-15-370).

Study participants were recruited by the Direction of the Quebec Ministry of Public Health in the region of Montérégie in collaboration with the researchers, municipalities, and community organizations of the city of Longueuil. Individuals who met at least one of the following four eligibility criteria related to heat vulnerability were recruited:

1. being 65 years of age or older.
2. having heart or lung medical conditions.
3. suffering from diabetes, kidney failure or neurological disorders.
4. having mental health issues.

The call for participation in this project was published in local newspapers and social media and advertised in municipal buildings. Meetings with prospective participants were held to present the research project and to explain the conditions of participation. Individuals could register for the research project in person at these meetings, online, or by phone or email. A total of 1811 individuals who met the eligibility criteria were ultimately recruited. Given that participation in this project was voluntary, the sample of this study was not random.

### Data collection

The first draft of the questionnaire was prepared after reviewing the relevant literature on the issue [6, 26] and was reviewed by 5 public health experts. The revised version was then pretested on 22 people who met the aforementioned eligibility criteria.

The data used in this study were collected by three successive phone surveys. The first survey took place in 2015 from June 25 to July 14. The survey collected data on attitudes, norms, perceived control, and intentions to adopt heat protection actions. Collecting data on the socioeconomic and demographic characteristics of the study participants was also one of the objectives of this survey. A response rate of 79.4% was obtained. The second survey was conducted immediately after the 2015 heatwave, which hit the city of Longueuil between 27 and 29 July. Its objective was to collect data on the adoption of heat protection behaviours by study participants who responded to the first survey. A response rate of 82.6% was achieved. The third survey was conducted in June 2016 and, like the first survey, aimed to collect data on attitudes, norms, perceived control, and intentions. This survey was intended for the respondents of the first survey, and a response rate of 54.8% was obtained. A rich database was therefore built that contained two repeated measures of attitudes, norms, perceived control, and intentions and one measure of the adoption of heatwave protection behaviours in addition to the sociodemographic characteristics of the study participants.

### Data analysis

Heatwave protection intentions and behaviours were measured on ordinal scales, as discussed above. Ordinal logit regressions were therefore used to measure their associations with their presumed determinants. Specifically, the fixed effects ordered logit model for panel data was used to test the effect of attitudes, norms, and perceived control on the intention to adopt heat protection behaviours [27]. Data from the first and third surveys were used for this purpose. The fixed effects ordered logit model enables the control of unobservable confounding factors of study participants that are constant over time. As this design reduces the risk of endogeneity, it lends more credibility to causal inferences [28, 29]. To the best of our knowledge, no previous study has used this method to test the effects of attitudes, norms, and perceived control on the intention to adopt heatwave protection behaviours.

In this study, it was not possible to use the fixed effect model to test the effects of intentions and perceived control on the adoption of heat protection behaviours given that only one measurement of behaviours was completed. Instead, an ordered logit model for cross-sectional data was implemented. To mitigate the risk of endogeneity, the observable characteristics of the study participants were controlled for. Moreover, data on attitudes, norms, perceived control and intentions were collected in the first survey, and data on behaviour were collected in the second survey, as recommended for studies using the TPB [19, 20]. While this design does not provide strong protection against unobservable confounding factors compared to the fixed effect design, it has the advantage of attenuating consistency biases [21].

## Study results

### Descriptive statistics

Table 1 presents the sociodemographic characteristics of the study participants using the data of the first survey. The sample was composed mainly of women (72.5%), aged 65 years and older (81.1%), and born in Canada (93.6%). The data also indicate that 30.1% of the study participants had a university degree, and 71.9% had an annual gross family income less than CAD$50,000. Concerning health conditions, 73.4% of the respondents suffered from at least one of the following chronic diseases associated with vulnerability to hot weather: heart disease, diabetes, kidney failure, respiratory disease, and neurological disorders. Compared to census data on the population of Longueuil, women and elderly individuals were overrepresented in the sample of this study.

**Table 1:** Sample characteristics (*n* = 1338)

Variable	Percentage
Women	72.5%
Chronic diseases	73.4%
Age: 18–64 years	18.9%
Age: 65–74 years	44.3%
Age: 75 years or over	36.8%
Income < 25k	39.1%
Income between 25 and 50k	32.8%
Income more than 50k	28.3%
University diploma	30.1%
Immigrants	6.4%

Data source: First survey

Table 2 shows that the study participants had very positive attitudes towards heat protection behaviours. In fact, 95.9% and 95.6% of respondents considered drinking water and reducing physical effort, respectively, to be somewhat useful or totally useful for protecting themselves from the heat. These rates were 88.7% for frequenting cool or air-conditioned places, 84.8% for taking cool showers and baths and 88.9% for using air conditioning systems.

**Table 2:** Attitudes towards heatwave protection behaviours

Behaviours	Totally useless	Somewhat useless	Neutral	Somewhat useful	Totally useful
Drinking more water than usual	0.5%	1.1%	2.5%	13.1%	82.8%
Reducing physical effort	0.6%	0.8%	2.9%	15.7%	80.1%
Spending time in cool or air-conditioned places	4.4%	2.6%	4.3%	16.2%	72.5%
Using air conditioner	3.4%	2.2%	5.5%	16.7%	72.2%
Taking cool showers or baths	3.9%	2.3%	9.03%	22.9%	61.9%

Data source: First survey

The statistics on prescriptive norms presented in Table 3 indicate that a strong majority of respondents believed that most of the people who were significant to them thought that they should protect themselves from the heat (93.8%) or would advise them to do so (84.7%). The social context thus seems to support and encourage individuals to protect themselves from heatwaves.

**Table 3:** Prescriptive norms related to heatwave protection behaviours

Measurement scale	1	2	3	4	5
Most people significant to you think you should protect yourself from heat (1: totally disagree to 5: totally agree)	0.7%	1.0%	4.4%	14.6%	79.2%
Most people significant to you would advise you to protect yourself from heat (1: very improbable to 5: very probable)	3.5%	4.5%	7.4%	21.1%	63.6%

Data source: First survey

Concerning perceived control, the implementation of heat protection behaviours did not seem to be challenging for most of the study participants, as shown in Table 4.

**Table 4:** Perceived control of heatwave protection behaviours

Measurement scale	Very difficult	Somewhat difficult	Neutral	Somewhat easy	Very easy
Drink more water than usual	1.8%	3.6%	5.3%	14.4%	75.1%
Reduce physical effort	2.4%	2.0%	5.9%	18.3%	71.5%
Spend time in cool or air-conditioned places	6.0%	6.2%	10.2%	18.6%	59.0%
Use air conditioner	1.5%	0.8%	2.3%	12.9%	82.6%
Take cool showers or baths	3.9%	2.7%	5.9%	16.3%	71.15%

Data source: First survey

Table 5 indicates that in the case of a heatwave, a high proportion of respondents had the intention to drink more water than usual (88.4%), reduce physical effort (91.3%), use air conditioners (88.4%) and take cool showers or baths (76.8%). However, just over half of the respondents expressed their intention to frequent cool or air-conditioned places (52.9%).

**Table 5:** Intentions to adopt heat protection behaviours

Measurement scale	Very improbable	Somewhat Improbable	Neutral	Somewhat probable	Very probable
Drink more water than usual	3.9%	1.9%	5.8%	16.6%	71.8%
Reduce physical effort	2.3%	1.7%	4.7%	15.7%	75.6%
Spend time in cool or air-conditioned places	17.6%	13.3%	16.3%	17.7%	35.2%
Use air conditioner	3.6%	1.9%	5.8%	12.3%	76.1%
Take cool showers or baths	7.4%	4.9%	11%	17.9%	58.9%

Data source: First survey

The second survey collected data on the behaviours of the study participants during the 2015 heatwave from 27 to 29 July. We detected significant differences across heat protection behaviours. A strong majority of respondents reported that during this episode, they took cool baths or showers (69.3%), increased their consumption of water (67%), used air conditioners more (77.4%), and reduced their physical effort (64.7%) (Table 6). In contrast, only 40.5% of the respondents visited cool or air-conditioned places, and 32.4% of those respondents reported doing so more often than usual. It is also interesting to note that a significant portion of the study participants did not change their behaviours during this episode, as indicated in the fourth column of Table 6.

**Table 6:** Adoption of heatwave protection behaviours

Measurement scale	Much less than usual	Slightly less than usual	As much as usual	Slightly more than usual	Much more than usual
Drank water	0%	0.4%	32.7%	34.7%	32.3%
Exerted physical effort	31.9%	32.8%	31%	3.1%	1.4%
Spent time in cool or air-conditioned places	2.1%	4.2%	61.4%	25.9%	6.5%
Used air conditioner	0.2%	0.6%	31.1%	22.8%	54.6%
Took cool showers or baths	0%	0.1%	56.1%	33.4%	10.4%

Data source: Second survey

### Drivers of intentions to adopt heatwave protection behaviours

As intentions to adopt heat protection behaviours were measured on a 5-point scale, ordinal logit models for panel data were used to test the effect of attitudes, norms and perceived control on this variable [27]:

$$\eqalign{& {\rm{Logit}}\,{\rm{[Pr}}\,{\rm{(}}{{\rm{Y}}_{{\rm{it}}}}\,{\rm{ > }}\,{\rm{S}}\,{\rm{|}}\,{{\rm{\alpha }}_{\rm{i}}}{\rm{,}}\,{{\rm{A}}_{{\rm{it}}}}{\rm{,}}\,{{\rm{N}}_{{\rm{it}}}}{\rm{,}}\,{{\rm{C}}_{{\rm{it}}}}{\rm{,}}\,{{\rm{T}}_{\rm{t}}}{\rm{)]}}\cr & {\rm{ = }}\,{{\rm{\alpha }}_{\rm{i}}}\,{\rm{ + }}\,{{\rm{\beta }}_{\rm{1}}}{{\rm{A}}_{{\rm{it}}}}\,{\rm{ + }}\,{{\rm{\beta }}_{\rm{2}}}{{\rm{N}}_{{\rm{it}}}}\,{\rm{ + }}\,{{\rm{\beta }}_{\rm{3}}}{{\rm{C}}_{{\rm{it}}}}\,{\rm{ + }}\,{{\rm{\beta }}_{\rm{t}}}{{\rm{T}}_{\rm{t}}}\, - \,{{\rm{K}}_{\rm{s}}} \cr} $$

Where:

Y_it_: intention of individual i expressed in period t to adopt heat protection behaviours;

S: category of the ordinal variable Y (intention has 5 ordered categories);

K_S_: parameter corresponding to category S;

α_i_: fixed effect specific to individual i;

A_it_: attitudes of individual i in period t;

N_it_: perception of norms by individual i in period t;

C_it_: perceived control by i in period t;

T_t_: data collection period (0 = summer 2015, 1 = summer 2016).

The fixed-effects panel model enables us to control for confounding factors that are constant over time (α_i)_, such as respondents’ sex or ethnicity. Moreover, introducing the period variable into the model makes it possible to control for the time effect. However, this method is vulnerable to unobservable differences between study participants that vary over time (α_it)_, such as changes in the health status of respondents between the two measurement points or in the subjective factors not accounted for by the TPB. The data from the first survey (summer 2015) and the third survey (summer 2016) were used to implement the panel fixed effect logit model.

The data were analysed using the *feologit* command published in the Stata Journal in 2020 with the odds ratio option [29]. The results of this analysis are presented in Table 7. The odds ratios of attitudes and perceived control variables were greater than one and statistically significant in all models, suggesting that these variables reinforced the intention to adopt heat protection behaviours. Regarding the effects of norms, the odds ratio of this variable was greater than one and was statistically significant according to two models; however, in three models, no significant association with heat protection intentions was found. Attitudes and perceived control and, to a lesser extent, prescriptive norms thus behaved as predicted by the TPB. The analysis also suggested that their effects were not uniform across heat protection behaviours. In fact, the odds ratios varied between 1.53 and 4.02 for attitudes, 1.76 and 2.25 for perceived control and 1.10 and 2 for norms. In addition to differences across behaviours, attitudes and perceived control seemed to have a greater influence on intentions than prescriptive norms.

**Table 7:** The effect of attitudes, norms and perceived control on the intention to adopt heatwave protection behaviours

Variables	Drinking water	Reducing physical effort	Taking cool baths and showers	Visiting cool or air-conditioned places	Using AC
Attitudes	1.83*** (0.43)	2.88*** (0.81)	2.37*** (0.37)	1.53*** (0.16)	4.02*** (1.23)
Norms	2.00*** (0.44)	1.39* (0.27)	1.85*** (0.39)	1.19 (0.18)	1.10 (0.27)
Perceived control	1.84*** (0.31)	2.03*** (0.40)	2.38*** (0.42)	1.76*** (0.22)	2.25*** (0.52)
Period	0.96 (0.16)	0.94 (0.18)	1.35* (0.18)	1.35** (0.18)	1.63** (0.36)
N	476	416	546	692	284
Wald chi2 Pseudo R square	41.02*** 0.19	34.89*** 0.24	61.87*** 0.37	45.93*** 0.14	35.79*** 0.33

Odds ratios are reported

Robust standard errors in parentheses

***: *p* < 0.01; **: *P* < 0.05; *: *P* < 0.10

As mentioned above, some extensions of the TPB suggest that perceived control moderates the effects of attitudes and norms on intentions [14]. The interaction terms of perceived control with attitudes and norms were introduced to test this assumption. To reduce collinearity between the variables and their interaction terms, the mean-centred values of the independent variables (subtracting the means from the variables) were used in regression analyses (Tabachnick and Fidell, 2001). As indicated in Table 8, only the interaction term of the variable of perceived control of reducing physical effort with the variable of prescriptive norms was statistically significant, but it was inconsistent with the prediction of the TPB. The evidence thus suggests that perceived control does not significantly moderate the effect of norms and attitudes on the intention to adopt heat protection behaviours.

**Table 8:** Testing the moderating effect of perceived control on heatwave protection intentions

Variables	Drinking water	Reducing physical efforts	Taking cool baths and showers	Visiting cool or air-conditioned places	Using the AC
Attitudes	1.96** (0.55)	3.21*** (1.01)	2.37*** (0.38)	1.52*** (0.16)	3.71*** (1.24)
Perceived control	1.93*** (0.31)	2.64*** (0.58)	2.38*** (0.45)	1.76*** (0.23)	2.41*** (0.57)
Norms	2.12*** (0.47)	1.75** (0.38)	1.88*** (0.39)	1.13 (0.17)	1.07 (0.27)
Attitudes* control	1.19 (0.16)	1.12 (0.15)	0.99 (0.13)	1.01 (0.07)	1.27 (0.37)
Norm * control	0.85 (0.22)	1.78*** (0.36)	1.10 (0.18)	1.19 (0.13)	1.90 (0.75)
Period	0.96 (0.16)	099 (0.20)	1.36* (0.24)	1.35** (0.18)	1.63** (0.36)
N Wald chi2 Pseudo R square	476 48.07*** 0.20	416 40.43*** 0.29	546 62.67*** 0.37	692 46.59*** 0.14	284 38.52*** 0.35

Odds ratios are reported

Robust standard errors in parentheses

***: *p* < 0.01; **: *P* < 0.05; *: *P* < 0.1

The panel fixed effects model exploits within-individual variance to estimate regression coefficients. In the case of the TPB, this can be an important challenge as attitudes, norms and perceived control are belief-based constructs and beliefs, particularly core beliefs, are presumed to be relatively stable over time [30]. This would be an issue in this study if some categories of respondents did not change their attitudes and perceptions and, consequently, if the study results were not applicable to their situation. To explore this possibility, the percentages of respondents whose attitudes, perceived control and perceived norms remained unchanged between the two measurement points are reported in Tables 9, 10 and 11, respectively. The data indicate that the stability of the constructs of the TPB is not an issue for this study as all categories of respondents reported significant changes in their attitudes and perceptions between the two periods. However, the frequency of this change was not uniform across categories, with respondents reporting maximum scores indicating the highest percentages of unchanged attitudes and perceptions.

**Table 9:** Percentage of respondents whose attitudes did not change between the first and second periods

Response categories	Drinking water	Reducing physical effort	Visiting cool or air-conditioned places	Taking cool baths and showers	Using the AC
Not at all useful	14.25	0	17.14	29.17	31.25
Not useful	0	0	4.26	8.82	25
Somewhat useful	20.00	12.00	12.12	18.28	29.17
Useful	15.23	24.56	16.77	27.36	24.53
Very useful	80.13	86.43	82.99	73.33	82.25
All categories	64.89	64.89	50.47	46.32	68.07

**Table 10:** Percentage of respondents whose perceived control did not change between the first and second periods

Response categories	Drinking water	Reducing physical effort	Visiting cool or air-conditioned places	Taking cool baths and showers	Using the AC
Very difficult	20.00	44.44	32.14	50.00	0
Difficult	6.25	19.5	22.73	13.33	-
Somewhat difficult	17.39	18.92	31.94	14.04	16.67
Easy	19.15	26.67	29.14	25.45	16.67
Very easy	82.53	79.37	76.92	80	87.33
All categories	64.06	61.55	58.07	52.27	71.83

**Table 11:** Percentage of respondents whose perceived norms did not change between the first and second periods

Response categories	Norm 1	Norm 2
1	0.00	5.88
2	0.00	6.67
3	9.68	8.62
4	29.41	31.52
5	85.34	71.13
All categories	68.81	50.84

Norm 1: from 1 = totally disagree to 5 = totally agree

Norm 2: from 1 = very improbable to 5 = very probable

### Factors affecting the adoption of heat protection behaviours

The associations of the intention and perceived control with the frequency of adopting heat protection behaviours according to the ordinal logit model for cross-sectional data are presented in Table 12. In this analysis, the sex, education, age and health conditions of the study participants were used as control variables.

The results showed that the odds ratios of intentions were greater than one and were statistically significant for four out of five behaviours. Individuals with strong intentions were therefore more likely to increase the frequency of adopting heat protection actions. In contrast, the odds ratios of perceived control were not statistically significant in four models, suggesting that this variable did not have a direct influence on heat protection behaviours. The positive associations between intentions and heatwave protection behaviours were predicted by the TPB and were convergent with the results of empirical studies on this topic. In fact, the only available study that explicitly used the TPB to analyse heatwave adaptation found a positive effect of intentions on behaviours [10]. Likewise, two meta-analyses of studies in the field of public health that used prospective designs and controlled for past behaviours showed positive associations between intentions and behaviours [18, 22]. Contrary to our results, however, these studies showed that perceived control had a direct effect on behaviours in addition to its indirect effect through intentions.

Regarding the control variables, the TPB presumes that the effects of background factors on behaviours are mediated by attitudes, norms, perceived control and intentions. The odds ratios of the control variables, however, suggest that sex, education, age and respiratory diseases have direct effects on behaviours.

**Table 12:** The association between intentions and perceived control and heat protection behaviours

Variables	Drinking water	Reducing physical effort	Taking cool baths and showers	Visiting cool and air-conditioned places	Using the AC
Intentions	1.60*** (0.13)	1.26*** (0.10)	1.56*** (0.10)	1.49*** (0.08)	1.02 (0.08)
Perceived control	1.12 (0.08)	1.01 (0.07)	1.16** (0.09)	1.04 (0.07)	1.03 (0.13)
Sex (1 = man, 0 = women))	0.67*** (0.09)	0.60*** (0.08)	0.94 (0.13)	0.82 (0.12)	0.63*** (0.09)
Education	0.97 (0.05)	1.03 (0.06)	0.85*** (0.05)	1.03 (0.06)	1.18** (0.07)
Age > 75 years	0.80* (0.11)	0.72** (0.09)	0.85 (0.11)	0.91 (0.13)	0.83 (0.12)
Heart disease	1.18 (0.15)	1.09 (0.14)	0.99 (0.12)	0.97 (0.13)	1.17 (0.16)
Respiratory disease	1.26* (0.17)	1.61*** (0.22)	1.03 (0.14)	0.75* (0.12)	1.21 (0.19)
Diabetes	0.97 (0.14)	1.21 (0.17)	1.30* (0.19)	1.08 (0.17)	0.92 (0.15)
Neurological disorder	0.73 (0.15)	0.91 (0.18)	1.11 (0.23)	1.29 (0.28)	0.99 (0.22)
N	970	958	963	935	781
LR chi2 Pseudo R square	79.50*** 0.04	53.36*** 0.02	120.42*** 0.05	89.06*** 0.04	20.28** 0.01

Odds ratios are reported

Standard errors in parentheses

***: *p* < 0.01; **: *P* < 0.05; *: *P* < 0.1

While the TPB is useful for understanding the effect of individuals’ subjectivity on heatwave protection behaviours, it fails to address the needs of public health policy-makers and professionals to obtain insight into the level of compliance with heatwave protection recommendations by individuals from different backgrounds, notably those who are vulnerable to heatwaves. This information is more relevant for the design of heat protection interventions that are based on objective characteristics than on subjective and less observable characteristics of the population. The relationship between heat protection behaviours and the factors associated with vulnerability to this hazard that were used to select the participants in this study were therefore reanalysed without accounting for the mediating effects of the TPB constructs. This analysis differed from the one presented in Table 12 because the odds ratios measure the total effects on behaviours, not only the direct effects of the background factors included in the regressions.

The results of this analysis are presented in Table 13. Notably, the odds ratios of individuals with heart disease, diabetes and neurological disorders were close to one and not statistically significant. These health conditions do not seem to influence heat protection behaviours. Individuals with respiratory diseases are more likely to reduce their physical effort during heatwaves, but they are less inclined to visit cool or air-conditioned places. Regarding the effect of age, the odds ratio for individuals older than 75 years was less than one according to four models (p value < 0.1), suggesting that seniors are less likely to adapt their behaviours to heatwaves than are individuals in the other age groups. The odds ratio of sex was statistically significant according to three models and was between 0.57 and 0.64, suggesting that men were less likely than women to protect themselves from heatwaves. Finally, the effect of education was not consistent across behaviours. Education seemed to decrease the odds ratio of taking cool showers and to increase the odds ratio of using air conditioners.

**Table 13:** Association between background conditions and heatwave protection behaviours

Variables	Drinking water	Reducing physical effort	Taking cool baths and showers	Visiting cool or air-conditioned places	Using the AC
Sex (1 = man, 0 = women))	0.64*** (0.08)	0.57*** (0.07)	0.83 (0.11)	0.82 (0.12)	0.63*** (0.09)
Education	1.00 (0.05)	1.08 (0.06)	0.83*** (0.04)	1.01 (0.06)	1.19*** (0.07)
Age > 75 years	0.77** (0.10)	0.73** (0.09)	0.80* (0.10)	0.78* (0.10)	0.84 (0.12)
Heart disease	1.10 (0.14)	1.13 (0.14)	0.96 (0.12)	0.97 (0.13)	1.16 (0.16)
Respiratory disease	1.12 (0.15)	1.58*** (0.21)	0.97 (0.13)	0.74** (0.11)	1.14 (0.17)
Diabetes	0.91 (0.13)	1.23 (0.17)	1.26 (0.18)	1.10 (0.17)	0.94 (0.15)
Neurological disorder	0.72* (0.14)	0.89 (0.17)	0.95 (0.20)	1.12 (0.25)	1.07 (0.23)
N	998	982	995	1000	818
LR chi2 Pseudo R square	20.11*** 0.01	42.21*** 0.02	21.45*** 0.01	11.94* 0.01	20.28*** 0.01

Odds ratios are reported

Standard errors in parentheses

***: *p* < 0.01; **: *P* < 0.05; *: *P* < 0.1

## Discussion

The fight against heatwaves is a shared responsibility between governments, citizens and the private sector [31]. This collective responsibility is notably salient in the case of heat warning systems and awareness campaigns, as the government is expected to provide relevant information in a timely fashion to help citizens react positively to weather hazards by adopting appropriate protective actions. Since these interventions use information as policy instruments to influence individuals’ behaviour, their effectiveness is largely conditional on the validity of the assumptions related to human behaviours. This situation highlights the importance of using well-established theoretical models to understand the factors that influence the ways in which individuals react to weather hazards [5, 11].

This study is one of the few studies to systematically use the TPB to identify the factors that explain the adoption of five protection behaviours that are commonly recommended to mitigate the adverse effects of heatwaves on population health. The analysis indicated that attitudes, prescriptive norms and perceived control had positive effects on intentions to adopt heat protection actions. Moreover, a positive association was found between intentions and heat protection behaviours. These results are consistent with the TPB and empirical studies that use this theory to predict actions undertaken by individuals to protect themselves from heatwaves [10] and health risks [20, 22] and to explain behaviours in general [18, 22, 32]. However, contrary to Barbara and Ajzen (2020), the assumption that perceived control moderates the effects of attitudes and prescriptive norms on intentions was not supported by the results of this study. The TPB thus contributes to understanding heatwave adaptation by stressing the importance of attitudes, norms and perceived control in choosing protection actions.

In addition to assessing the capacity of the TPB to explain heatwave adaptation, this study explored the associations between risk factors and the adoption of preventive measures. Compared with individuals in other social groups, men and elderly individuals were less likely to adjust their behaviours to heatwaves. Furthermore, health conditions associated with increased risks of heatwaves, namely, heart and respiratory diseases, diabetes, and neurological conditions, did not seem to have a notable influence on heat protection behaviours. These results are rather concerning given that individuals who are vulnerable to heatwaves have a greater need to adapt their behaviours to this weather hazard.

Regarding the policy implications of this study, in principle, heat warnings are used to alert the population about heatwaves, raise awareness of the associated health threats, and recommend health protection actions [3]. As this study has found that intentions to adopt heat protection behaviours are affected by individuals’ attitudes, perceived norms, and perceived control, awareness campaigns that target the key beliefs underlying these factors could improve the effectiveness of heat warnings [32]. The available evidence from different behavioural domains suggests that the underlying key beliefs of the TPB are pliable and therefore susceptible to influence by public health interventions [32]. However, as little is known about this topic in heat protection behaviours, further research is needed to address this question. Similarly, this study suggests that heat warnings would be more effective when they take into account users’ ability to adopt the recommended behaviours. The findings also reveal the need to take action to encourage men and individuals who are vulnerable to hot weather to adopt the heat protection actions recommended by public health authorities and experts.

Among the limitations of this study, the TPB distinguishes between prescriptive and descriptive norms, but the effect of descriptive norms was not analysed in this study. Moreover, the estimations of the effects of intentions and perceived control on behaviours would have been more consistent if repeated measures of heat protection behaviours were taken. Finally, the participants in this study were not randomly selected, which limits the generalizability of the findings. Notwithstanding these limitations, this study has the advantage of using longitudinal and prospective designs to test the effects of attitudes, norms, and intentions on heat protection behaviours. Compared to most studies on this issue, this study is better able to control for unobserved fixed confounding factors when testing the effect of attitudes, norms and perceived control on intentions and to reduce consistency biases when analysing the factors that influence the adoption of heat protection behaviours.

## Conclusion

Using the ordinal panel fixed effect model, this study found firm evidence supporting the assumption that attitudes, prescriptive norms, and perceived control have positive effects on the intention to adopt heat protection behaviours. Moreover, the analysis rejects the assumption that the effects of attitudes and norms are moderated by the perception of control. A cross-sectional ordinal logit model was used to analyse the determinants of the adoption of heat protection behaviours. The results suggest the presence of positive associations between intentions and behaviours. However, old age and health conditions that are associated with vulnerability to heatwaves were not found to improve protective behaviours.

## Data Availability

The datasets used and/or analysed during the current study are available from the corresponding author upon reasonable request.

## Abbreviations

TPB: Theory of planned behaviour

## References

[1] Bustinza R, Lebel G, Gosselin P, Bélanger D, Chebana F (2013). Health impacts of the July 2010 heat wave in Québec, Canada. BMC Public Health.

[2] Henderson SB, McLean KE, Lee MJ, Kosatsky T (2022). Analysis of community deaths during the catastrophic 2021 heat dome: early evidence to inform the public health response during subsequent events in greater Vancouver, Canada. Environ Epidemiol.

[3] Mehiriz K, Gosselin P, Tardif I, Lemieux MA (2018). The Effect of an Automated Phone Warning and Health Advisory System on Adaptation to High Heat Episodes and Health Services Use in vulnerable groups—evidence from a Randomized Controlled Study. Int J Environ Res Public Health.

[4] Erens B, Williams L, Exley J, Ettelt S, Manacorda T, Hajat S (2021). Public attitudes to, and behaviours taken during, hot weather by vulnerable groups: results from a national survey in England. BMC Public Health.

[5] Swim JK, Stern PC, Doherty TJ, Clayton S, Reser JP, Weber EU (2011). Psychology’s contributions to understanding and addressing global climate change. Am Psychol.

[6] Bélanger D, Abdous B, Valois P, Gosselin P, Sidi EAL (2016). A multilevel analysis to explain self-reported adverse health effects and adaptation to urban heat: a cross-sectional survey in the deprived areas of 9 Canadian cities. BMC Public Health.

[7] Beckmann S, Hiete M (2020). Predictors Associated with Health-Related Heat Risk Perception of Urban Citizens in Germany. Int J Environ Res Public Health.

[8] Akompab D, Bi P, Williams S, Grant J, Walker I, Augoustinos M (2013). Heat waves and climate change: applying the Health Belief Model to identify predictors of risk perception and adaptive behaviours in Adelaide, Australia. Int J Environ Res Public Health.

[9] Beckmann SK, Hiete M, Schneider M, Beck C (2021). Heat adaptation measures in private households: an application and adaptation of the protective action decision model. Humanit Soc Sci Commun.

[10] Valois P, Talbot D, Bouchard D, Renaud JS, Caron M, Canuel M (2020). Using the theory of planned behavior to identify key beliefs underlying heat adaptation behaviors in elderly populations. Popul Environ.

[11] Mehiriz K, Gosselin P. The Effect of Perceived threats and Response Efficacy on Adaptation to Smog: an instrumental variables design. Risk Anal. 2021;risa.13814. 10.1111/risa.13814 34424564

[12] Gifford R (2014). Environmental psychology matters. Annu Rev Psychol.

[13] Richard L, Kosatsky T, Renouf A (2011). Correlates of hot day air-conditioning use among middle-aged and older adults with chronic heart and lung diseases: the role of health beliefs and cues to action. Health Educ Res.

[14] La Barbera F, Ajzen I (2020). Control interactions in the theory of planned behavior: rethinking the role of subjective norm. Eur J Psychol.

[15] Ajzen I (2015). The theory of planned behaviour is alive and well, and not ready to retire: a commentary on Sniehotta, Presseau, and Araújo-Soares. Health Psychol Rev.

[16] Ajzen I, Fishbein M (1969). The prediction of behavioral intentions in a choice situation. J Exp Soc Psychol.

[17] Ajzen I (1991). The theory of planned behavior. Organ Behav Hum Decis Process.

[18] McEachan RRC, Conner M, Taylor NJ, Lawton RJ (2011). Prospective prediction of health-related behaviours with the theory of Planned Behaviour: a meta-analysis. Health Psychol Rev.

[19] Bleakley A, Hennessy M, Fishbein M, Jordan A (2011). Using the integrative model to explain how exposure to sexual media content influences adolescent sexual behavior. Health Educ Behav.

[20] Matterne U, Diepgen TL, Weisshaar E (2011). A longitudinal application of three health behaviour models in the context of skin protection behaviour in individuals with occupational skin disease. Psychol Health.

[21] Sussman R, Gifford R (2019). Causality in the theory of Planned Behavior. Pers Soc Psychol Bull.

[22] Hagger MS, Hamilton K (2023). Longitudinal tests of the theory of planned behaviour: a meta-analysis. Eur Rev Soc Psychol.

[23] Hajat S, O’Connor M, Kosatsky T (2010). Health effects of hot weather: from awareness of risk factors to effective health protection. The Lancet.

[24] Ban J, Shi W, Cui L, Liu X, Jiang C, Han L (2019). Health-risk perception and its mediating effect on protective behavioral adaptation to heat waves. Environ Res.

[25] Li J, Xu X, Ding G, Zhao Y, Zhao R, Xue F (2016). A cross-sectional study of Heat Wave-related knowledge, attitude, and practice among the Public in the Licheng District of Jinan City, China. Int J Environ Res Public Health.

[26] Kalkstein AJ, Sheridan SC (2007). The social impacts of the heat–health watch/warning system in Phoenix, Arizona: assessing the perceived risk and response of the public. Int J Biometeorol.

[27] Baetschmann G, Staub KE, Winkelmann R (2015). Consistent estimation of the fixed effects ordered Logit Model. J R Stat Soc Ser a Stat Soc.

[28] Mehiriz K (2016). The impacts of intergovernmental grants on municipal infrastructure: evidence from the Canada-Quebec infrastructure works 2000 program. Eval Program Plann.

[29] Baetschmann G, Ballantyne A, Staub KE, Winkelmann R (2020). Feologit: a new command for fitting fixed-effects ordered logit models. Stata J.

[30] Sabatier PA (1988). An Advocacy Coalition Framework of Policy Change and the role of policy-oriented learning therein. Policy Sci.

[31] United Nations. Sendai Framework for Disaster Risk Reduction 2015–2030.:37.

[32] Steinmetz H, Knappstein M, Ajzen I, Schmidt P, Kabst R (2016). How effective are Behavior Change interventions based on the theory of Planned Behavior? A three-level Meta-analysis. Z Für Psychol.

